# A self-amplifying loop of TP53INP1 and P53 drives oxidative stress-induced apoptosis of bone marrow mesenchymal stem cells

**DOI:** 10.1007/s10495-023-01934-1

**Published:** 2024-03-16

**Authors:** Fanchao Li, Fei Zhang, Tao Wang, Zhihong Xie, Hong Luo, Wentao Dong, Jian Zhang, Chao Ren, Wuxun Peng

**Affiliations:** Department of Orthopedics and Traumatology, The Affiliated Hospital of Guizhou Medical University, Guiyang, 550004 Guizhou China

**Keywords:** Bone marrow mesenchymal stem cells, Oxidative stress, Apoptosis, Tumor suppressor p53, Tumor protein p53 inducible nuclear protein 1

## Abstract

Bone marrow mesenchymal stem cell (BMSC) transplantation is a promising regenerative therapy; however, the survival rate of BMSCs after transplantation is low. Oxidative stress is one of the main reasons for the high apoptosis rate of BMSCs after transplantation, so there is an urgent need to explore the mechanism of oxidative stress-induced apoptosis of BMSCs. Our previous transcriptome sequencing results suggested that the expression of P53-induced nuclear protein 1 (TP53INP1) and the tumor suppressor P53 (P53) was significantly upregulated during the process of oxidative stress-induced apoptosis of BMSCs. The present study further revealed the role and mechanism of TP53INP1 and P53 in oxidative stress-induced apoptosis in BMSCs. Overexpression of TP53INP1 induced apoptosis of BMSCs, knockdown of TP53INP1 alleviated oxidative stress apoptosis of BMSCs. Under oxidative stress conditions, P53 is regulated by TP53INP1, while P53 can positively regulate the expression of TP53INP1, so the two form a positive feedback loop. To clarify the mechanism of feedback loop formation. We found that TP53INP1 inhibited the ubiquitination and degradation of P53 by increasing the phosphorylation level of P53, leading to the accumulation of P53 protein. P53 can act on the promoter of the *TP53INP1* gene and increase the expression of *TP53INP1* through transcriptional activation. This is the first report on a positive feedback loop formed by TP53INP1 and P53 under oxidative stress. The present study clarified the formation mechanism of the positive feedback loop. The TP53INP1–P53 positive feedback loop may serve as a potential target for inhibiting oxidative stress-induced apoptosis in BMSCs.

**Supplementary Information:**

The online version contains supplementary material available at 10.1007/s10495-023-01934-1.

## Introduction

Regenerative medicine, as a multidisciplinary field with the goal of promoting the repair and regeneration of tissues and organs, has attracted extensive attention in recent years [1]. Bone marrow mesenchymal stem cells (BMSCs) are one of the most important seed cells for stem cell therapy in regenerative medicine. BMSCs have shown good repair effects in various degenerative diseases, animal models, and human clinical experiments [2–4]. Despite the great potential of BMSC-based stem cell therapy, transplanted BMSCs have a high apoptosis rate in the lesion area or tissue, which greatly limits the efficacy of BMSC transplantation [5]. Studies have found that oxidative stress injury is the main cause of BMSC apoptosis [6]. At present, oxidative stress-induced apoptosis is mainly inhibited by scavenging excessive reactive oxygen species (ROS) and reducing ROS production. Zhang et al. confirmed that PARK7 acts as a ROS scavenger and antioxidant to remove generated ROS by regulating the Nrf2 signaling pathway [7]. Wang et al. found that NMNAT3 inhibits the generation of ROS by increasing NAD^+^ levels, improving mitochondrial function [8]. Although these methods partially alleviate oxidative stress in cells, they do not block the already activated apoptotic pathways. So far, the mechanism of oxidative stress-induced apoptosis of BMSCs has not been finally determined. It is of great practical significance to explore the mechanism of oxidative stress-induced apoptosis of BMSCs and find new targets to inhibit stress-induced apoptosis of BMSCs.

Oxidative stress is caused by elevated ROS levels due to excessive intracellular ROS production or reduced ROS scavenging capacity. ROS attack lipids, membranes, proteins, and DNA, leading to structural and functional changes in cells, causing oxidative stress damage in cells and eventually leading to cell death [9]. Oxidative stress can cause apoptosis through both mitochondria-dependent and mitochondria-independent pathways [10]. Recent studies have found that the pathways controlling oxidative stress-induced apoptosis include the Fas, JNK, NF-κB, P53, and other signaling pathways [11–13].

The P53 signaling pathway exhibits cross-talk with the Fas, P38/MAPK, Wnt, PI3K/AKT, and other signaling pathways, which play important roles in redox homeostasis, apoptosis, cell cycle arrest, and so on[14–17]. In normal cells, P53 is inactive due to its interaction with MDM2. However, when BMSCs are exposed to oxidative stress, P53 expression is dramatically increased. It was found that oxidative stress stabilizes P53 by activating the JNK signaling pathway and inhibiting P53 degradation in human retinal pigment epithelial cells, and pretreatment with a JNK inhibitor abolished H_2_O_2_-induced P53 stabilization and activation [18]. Yang et al. found that doxorubicin promotes cardiomyocyte apoptosis by inducing P53 activation and ROS generation [19].

The activity and stability of P53 are mainly regulated by post-translational modifications such as ubiquitination, acetylation, phosphorylation, methylation, and SUMOylation [20]. It has been found that TP53INP1 is a regulator of P53 post-translational modifications [21]. TP53INP1 is an oxidative stress response protein that promotes *P53* gene expression by regulating post-translational modification of P53 in human osteosarcoma cells [22]. Meanwhile, the transcriptional induction of *TP53INP1* in response to oxidative stress is P53-dependent, and accumulated P53 was found to promote *TP53INP1* transcription in mouse embryonic fibroblasts [23]. Combined with previous studies that showed that the expression of P53 and TP53INP1 increased in BMSCs under oxidative stress, we hypothesized that TP53INP1 and P53 may form a positive feedback loop under oxidative stress to induce P53-mediated apoptosis. It is still unclear how TP53INP1 and P53 regulate oxidative stress-induced apoptosis in BMSCs and the relationship between them.

In the present study, we confirmed that overexpression of TP53INP1 and P53 in BMSCs induced apoptosis, and knockdown of P53INP1 and P53 under oxidative stress alleviated oxidative stress-induced apoptosis in BMSCs. A positive feedback loop between TP53INP1 and P53 was found and the formation mechanism of the positive feedback loop was revealed, which further promoted oxidative stress-induced apoptosis in BMSCs by inhibiting the PI3K/AKT and MEK/ERK1/2 signaling pathways. This study reveals the mechanism of oxidative stress-induced apoptosis of BMSCs, which is expected to provide a new target for reducing oxidative stress-induced apoptosis in BMSCs.

## Results

### Differential expression of TP53INP1 is associated with oxidative stress-induced apoptosis in BMSCs

**Fig. 1:**
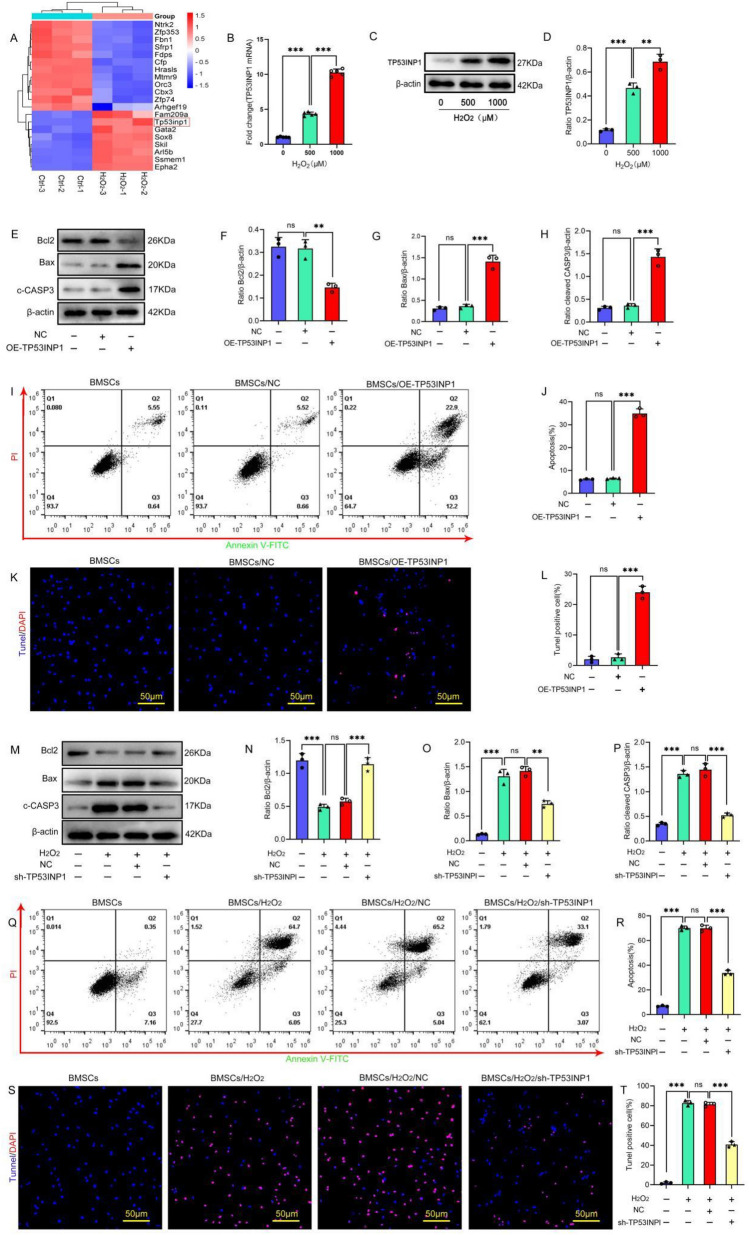
TP53INP1 mediates stress-induced apoptosis in BMSCs. **A** Heatmap showing differentially expressed genes. *TP53INP1* (red box) is the gene with the highest fold change value. **B** *TP53INP1* mRNA expression as determined by qPCR (*n* = 5). (C, D) TP53INP1 expression was detected by Western blot (*n* = 3). (E–H) The expression levels of Bcl2, Bax, and cleaved caspase-3 (C-CASP3) were determined by Western blot (*n* = 3). (I, J) Annexin V-FITC/PI staining was conducted to detect apoptosis (*n* = 3). (K, L) TUNEL/DAPI staining was used to determine the number of TUNEL-positive cells (*n* = 3). (M–P) The expression levels of Bcl2, Bax, and C-CASP3 were determined by Western blot (*n* = 3). (Q, R) Annexin V-FITC/PI staining was conducted to detect apoptosis (*n* = 3). (S, T) TUNEL/DAPI staining was used to determine the number of TUNEL-positive cells (*n* = 3). In **B, D, F–H, J, L, N–P, R, T**, data are described as mean ± standard deviation, and statistical analysis was performed using one-way ANOVA and the Tukey post hoc test

To investigate the potential mechanism of apoptosis in BMSCs under oxidative stress conditions, H_2_O_2_ was used to generate an oxidative stress cell injury model, followed by transcriptome analysis. The results showed that there were 152 differentially expressed genes (|log2(fold change)|≥ 1, *P* < 0.05), among which 102 genes were significantly upregulated and 50 genes were significantly downregulated after H_2_O_2_ treatment. The top 20 genes were selected for cluster analysis (Fig. 1A). *TP53INP1* was most significantly upregulated under oxidative stress. To verify the transcriptomic results, we treated BMSCs with different concentrations of H_2_O_2_ for 24 h and detected the expression level of *TP53INP1*. The results showed that the expression level of TP53INP1 increased with the increase of H_2_O_2_ concentration (Fig. 1B–D). These data suggest that the differential expression of *TP53INP1* may be related to stress-induced apoptosis in BMSCs.

First, we overexpressed TP53INP1 in BMSCs and examined the effect of TP53INP1 overexpression on BMSC apoptosis. The results showed that compared with the empty vector group, the expression of pro-apoptotic proteins Bax and cleaved caspase-3 in the TP53INP1 overexpression group was significantly upregulated, the expression of the anti-apoptotic protein Bcl2 was significantly downregulated, and the apoptosis rate of BMSCs was significantly increased (Fig. 1E–L). These results indicate that overexpression of TP53INP1 in BMSCs could induce BMSC apoptosis. Subsequently, we knocked out TP53INP1 in BMSCs under oxidative stress conditions to verify the effect of TP53INP1 knockout on oxidative stress-induced apoptosis in BMSCs. The results showed that H_2_O_2_ treatment increased the apoptosis rate of BMSCs compared with the NC group, and knockdown of TP53INP1 significantly reduced the apoptosis rate of BMSCs treated with oxidative stress (Fig. 1M–T). In conclusion, overexpression of TP53INP1 induced apoptosis and knockdown of TP53INP1 inhibited oxidative stress-induced apoptosis in BMSCs.

### P53 may be a downstream effector target of TP53INP1; overexpression of P53 induces apoptosis and knockout of P53 inhibits oxidative stress-induced apoptosis in BMSCs

**Fig. 2:**
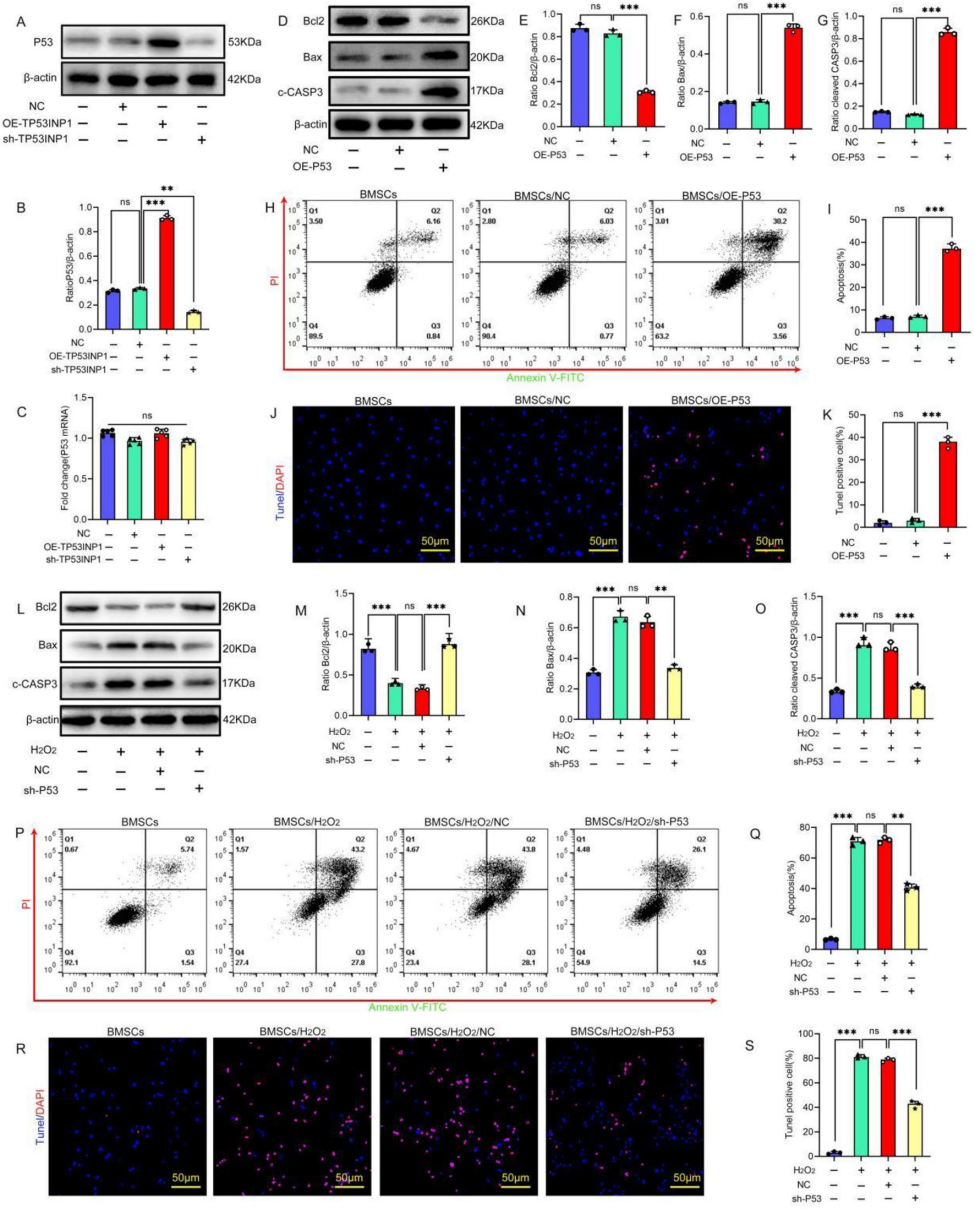
As a target of TP53INP1, P53 mediates stress-induced apoptosis in BMSCs. **A, B** P53 expression as determined by Western blot (*n* = 3). **C** *TP53* mRNA expression as determined by qPCR (*n* = 5). **D–G** The protein expression levels of Bcl2, Bax, and cleaved caspase-3 (C-CASP3) were detected by Western blot (*n* = 3). **H, I** Annexin V-FITC/PI staining was conducted to detect apoptosis (*n* = 3). **J, K** TUNEL/DAPI staining was used to determine the number of TUNEL-positive cells (*n* = 3). **L–O** The expression levels of Bcl2, Bax, and C-CASP3 were detected by Western blot (*n* = 3). **P, Q** Annexin V-FITC/PI staining was conducted to detect apoptotic cells (*n* = 3). **R, S** TUNEL/DAPI staining was conducted to determine the number of TUNEL-positive cells (*n* = 3). In **B, C, E–G, I, K, M–O, Q, S**, data are presented as mean ± standard deviation, and statistical analysis was performed using one-way ANOVA and the Tukey post hoc test

Studies have shown that TP53INP1 cooperates with P53 and plays an important role in the oxidative stress-induced activation of P53 [24]. However, whether TP53INP1 can affect the expression of P53 and the role of P53 in H_2_O_2_-induced apoptosis in BMSCs remains unclear. Therefore, we examined P53 expression in BMSCs with or without TP53INP1 overexpression. The results showed that P53 protein expression was upregulated in BMSCs with TP53INP1 overexpression and downregulated in BMSCs with TP53INP1 knockdown (Fig. 2A–B). There was no significant change in the mRNA expression of TP53, suggesting that TP53INP1 may regulate the expression of P53 protein at the post-transcriptional level (Fig. 2C). There was no significant change in the mRNA expression of *TP53*, suggesting that TP53INP1 may regulate the expression of P53 protein at the post-transcriptional level.

Next, we overexpressed P53 and then examined the effect of P53 on BMSC apoptosis. The results showed that compared with the empty vector group, the expression of pro-apoptotic proteins Bax and cleaved caspase-3 was significantly upregulated, the expression of the anti-apoptotic protein Bcl2 was significantly downregulated, and the apoptosis rate of BMSCs was significantly increased in the P53 overexpression group (Fig. 2D–K), indicating that overexpression of P53 could induce apoptosis in BMSCs. Subsequently, P53 was knocked out in BMSCs under oxidative stress conditions to verify the effect of P53 knockout on oxidative stress-induced apoptosis. The results showed that P53 knockout significantly reduced the apoptosis rate of BMSCs treated with oxidative stress compared with the empty vector group (Fig. 2L–S). In conclusion, P53 may be a downstream effector target of TP53INP1; overexpression of P53 induces apoptosis and knockdown of P53 inhibits oxidative stress-induced apoptosis in BMSCs.

### ***P53 is a downstream regulatory target of TP53INP1 in mediating H***_***2***_***O***_***2***_***-induced apoptosis in BMSCs***

**Fig. 3:**
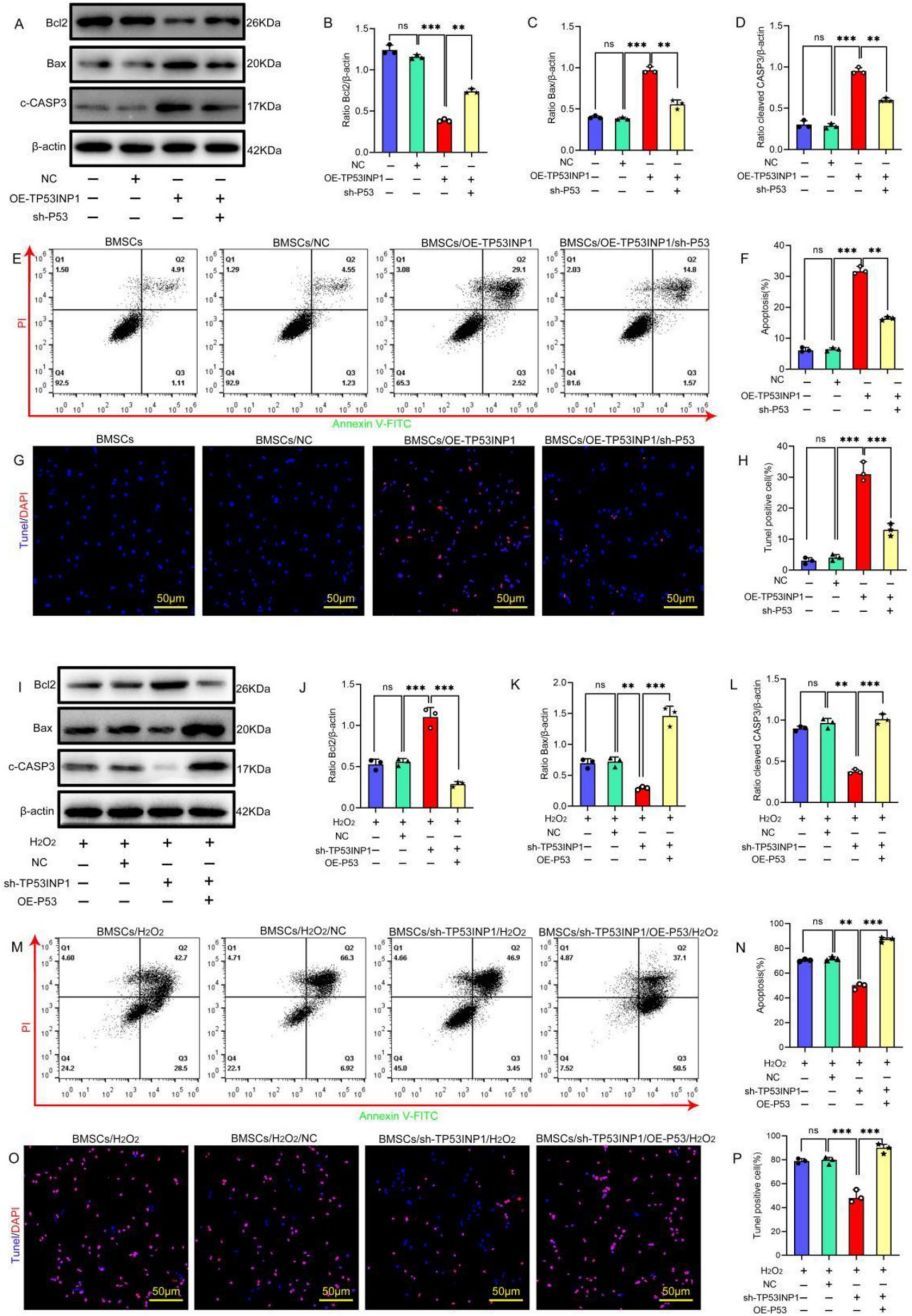
P53 is a downstream regulatory target of TP53INP1 in mediating H_2_O_2_-induced apoptosis in BMSCs **A, D** Western blot analysis was conducted to determine the expression levels of Bcl2, Bax, and cleaved caspase-3 (C-CASP3) (*n* = 3). **E, F** Annexin V-FITC/PI staining was conducted to detect apoptosis (*n* = 3). **G, H** TUNEL/DAPI staining was used to determine the number of TUNEL-positive cells (*n* = 3). **I–L** Western blot analysis was conducted to determine the expression levels of Bcl2, Bax, and C-CASP3 (*n* = 3). **M, N** FITC Annexin V/PI staining was conducted to detect apoptosis (*n* = 3). **O, P** TUNEL/DAPI staining was used to determine the number of TUNEL-positive cells (*n* = 3). In **B–D, F, H, J–L, N, P**, data are presented as mean ± standard deviation, and statistical analysis was performed using one-way ANOVA and the Tukey post hoc test

Upregulation of *TP53INP1* and *TP53* could induce apoptosis in BMSCs. Moreover, TP53INP1 could regulate the protein expression of P53. To further investigate whether TP53INP1 regulates stress-induced apoptosis in BMSCs through P53, first, we overexpressed TP53INP1 in BMSCs, which could significantly promote apoptosis. The protein expression of cleaved caspase-3 and Bax in BMSCs was significantly decreased and the protein expression of Bcl2 was significantly increased in P53 knockdown cells overexpressing TP53INP1. Moreover, the apoptosis rate of BMSCs was significantly decreased (Fig. 3A–H), indicating that knockdown of P53 was able to block the induction of apoptosis by overexpression of TP53INP1 in BMSCs. Next, we knocked down TP53INP1 in BMSCs under oxidative stress conditions; the results showed that knockdown of TP53INP1 significantly inhibited oxidative stress-induced apoptosis in BMSCs. However, overexpression of P53 in BMSCs with TP53INP1 knockdown resulted in increased oxidative stress-induced apoptosis (Fig. 3I–P). These results indicate that overexpression of P53 could attenuate the inhibitory effect of TP53INP1 knockdown on apoptosis in BMSCs. The above data suggest that P53 is a downstream regulatory target of TP53INP1 in mediating H_2_O_2_-induced apoptosis in BMSCs.

### Under oxidative stress, TP53INP1 and P53 form a positive feedback loop

**Fig. 4:**
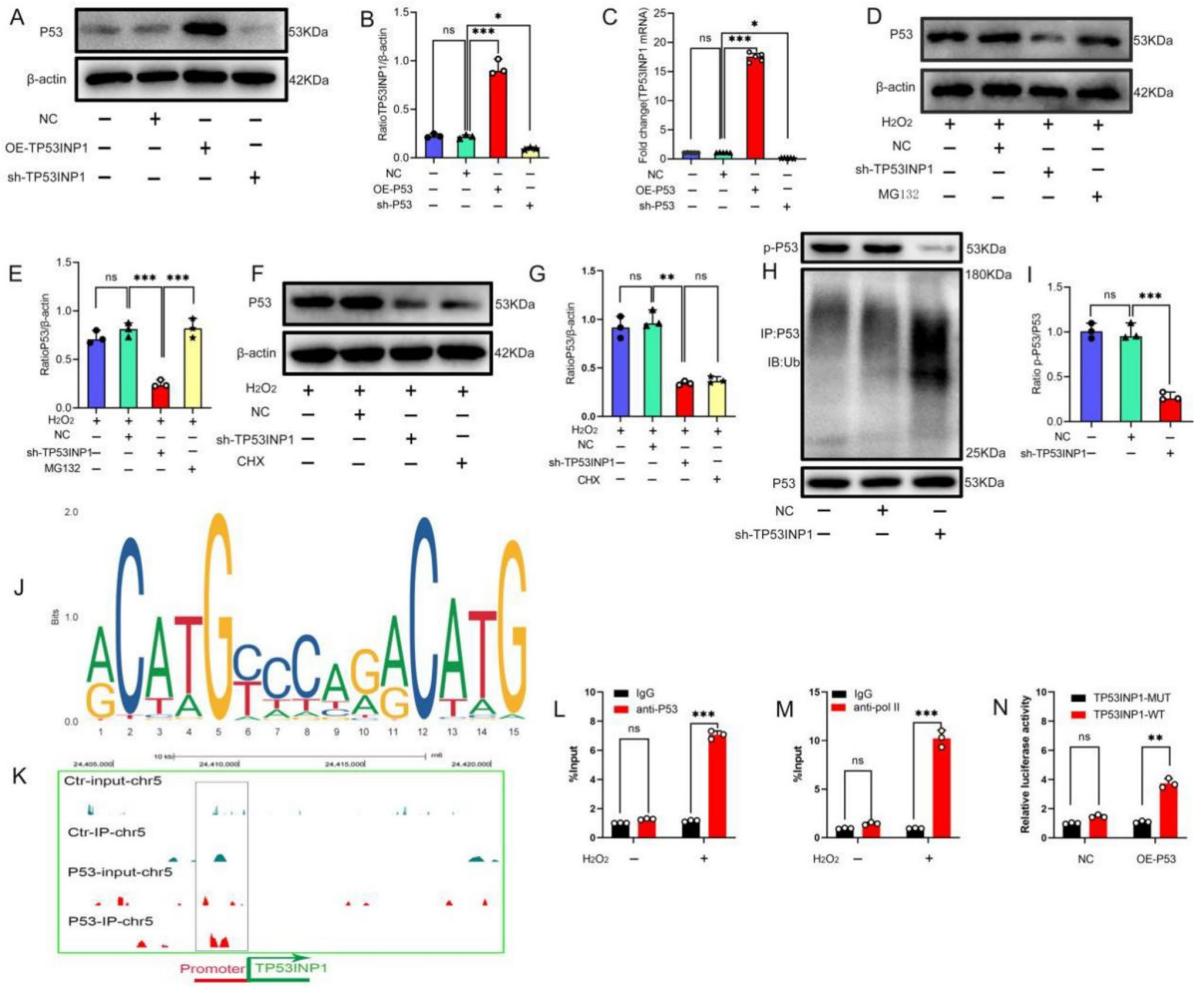
P53 and TP53INP1 form a feedback loop under oxidative stress conditions. **A, B** TP53INP1 expression as determined by Western blot (*n* = 3). (C) *TP53INP1* mRNA expression as determined by qPCR (*n* = 5). **D, E** Western blot analysis was conducted to determine the expression level of P53 after MG132 treatment (*n* = 3). **F**, **G** Western blot analysis of P53 expression after CHX treatment (*n* = 3). **H, I** Phosphorylation and ubiquitination levels of P53 as determined by Western blot analysis (*n* = 3). **J** Motif analysis results. **K** Visualization of P53 enrichment on chromosome 5. **L** ChIP-qPCR verified that P53 binds to the *TP53INP1* promoter. **M** ChIP-qPCR verified Pol II binding on the *TP53INP1* promoter. **N** The luciferase reporter assay verified that P53 promotes *TP53INP1* transcription. In **B, C, E, G, I, L–N**, data are presented as mean ± standard deviation, and statistical analysis was performed using one-way ANOVA and the Tukey post hoc test

P53 is a downstream target of TP53INP1, and TP53INP1 can regulate P53 protein expression (Figs. 2 and 3). It is well known that P53 is a transcription factor; once activated, P53 binds to the promoter regions of target genes to activate transcription [25]. We hypothesized that P53 regulates TP53INP1 expression, so we examined TP53INP1 expression in BMSCs with or without P53 overexpression. The results showed that the protein and mRNA expression levels of TP53INP1 in BMSCs with P53 knockdown were significantly decreased, while the protein and mRNA expression levels of TP53INP1 in BMSCs with P53 overexpression were significantly increased (Fig. 4A–C), suggesting that P53 may activate *TP53INP1* gene expression. Taken together, these results confirmed the existence of a positive feedback loop between TP53INP1 and P53.

Next, we further explored the mechanism of the formation of the positive feedback loop between TP53INP1 and P53. First, after knocking down TP53INP1, BMSCs were treated with the protein synthesis inhibitor cycloheximide (CHX) or the proteasome inhibitor (MG132) for 24 h. The results showed that CHX treatment had no effect on the P53 protein level, while MG132 treatment increased the P53 protein levels (Fig. 4D–G), indicating that TP53INP1 could not affect the production of P53, but affected the degradation of P53, thereby promoting the accumulation of P53. Phosphorylated P53 is the active form of P53, and its degradation depends on the ubiquitination degradation pathway. Subsequently, the phosphorylation and ubiquitination levels of P53 protein were detected, and the results showed that compared with the empty vector group, TP53INP1 knockdown significantly reduced the phosphorylation level and increased the ubiquitination level of P53 (Fig. 4H, I). Therefore, TP53INP1 increases the phosphorylation level of P53 and inhibits the ubiquitination and degradation of P53, leading to the accumulation of P53.

Our results show that P53 can regulate TP53INP1 mRNA and protein expression, and P53 acts as a transcription factor, so we further explored the mechanism by which P53 regulates TP53INP1. We predicted the existence of P53 binding sites (GCTTGACCTGGCTTG) in the *TP53INP1* promoter region using NCBI and Animal TFDB databases (Fig. 4J). P53 binding to the TP53INP1 promoter and the enrichment of RNA polymerase II in the *TP53INP1* promoter region were then verified by chromatin immunoprecipitation (ChIP) coupled with quantitative PCR (qPCR). The results showed that P53 interacted with the *TP53INP1* promoter and that the *TP53INP1* promoter region was significantly enriched in RNA polymerase II (Fig. 4K–M). Finally, we inserted the *TP53INP1* promoter sequence into pGL3 to construct a luciferase reporter plasmid, which was then transfected into BMSCs overexpressing P53. The results showed that compared with the NC group, overexpression of P53 did not affect luciferase activity in the TP53INP1-MUT group. Compared with the NC group, luciferase activity in the TP53INP1-WT group was significantly increased (Fig. 4N). These results further suggest that P53 could bind to the *TP53INP1* promoter to promote transcription, thereby regulating the expression of TP53INP1. In conclusion, there is a P53–TP53INP1 positive feedback loop under oxidative stress conditions. TP53INP1 inhibits the ubiquitination and degradation of P53 by increasing the phosphorylation level of P53 and promoting the accumulation of P53 protein. P53 binds to the *TP53INP1* promoter to promote transcription, thereby regulating the expression of TP53INP1. As such, a positive feedback loop is formed between the two to regulate apoptosis in BMSCs.

### P53 mediates oxidative stress-induced apoptosis in BMSCs by inhibiting the PI3K/AKT and MEK/ERK1/2 pathways

**Fig. 5:**
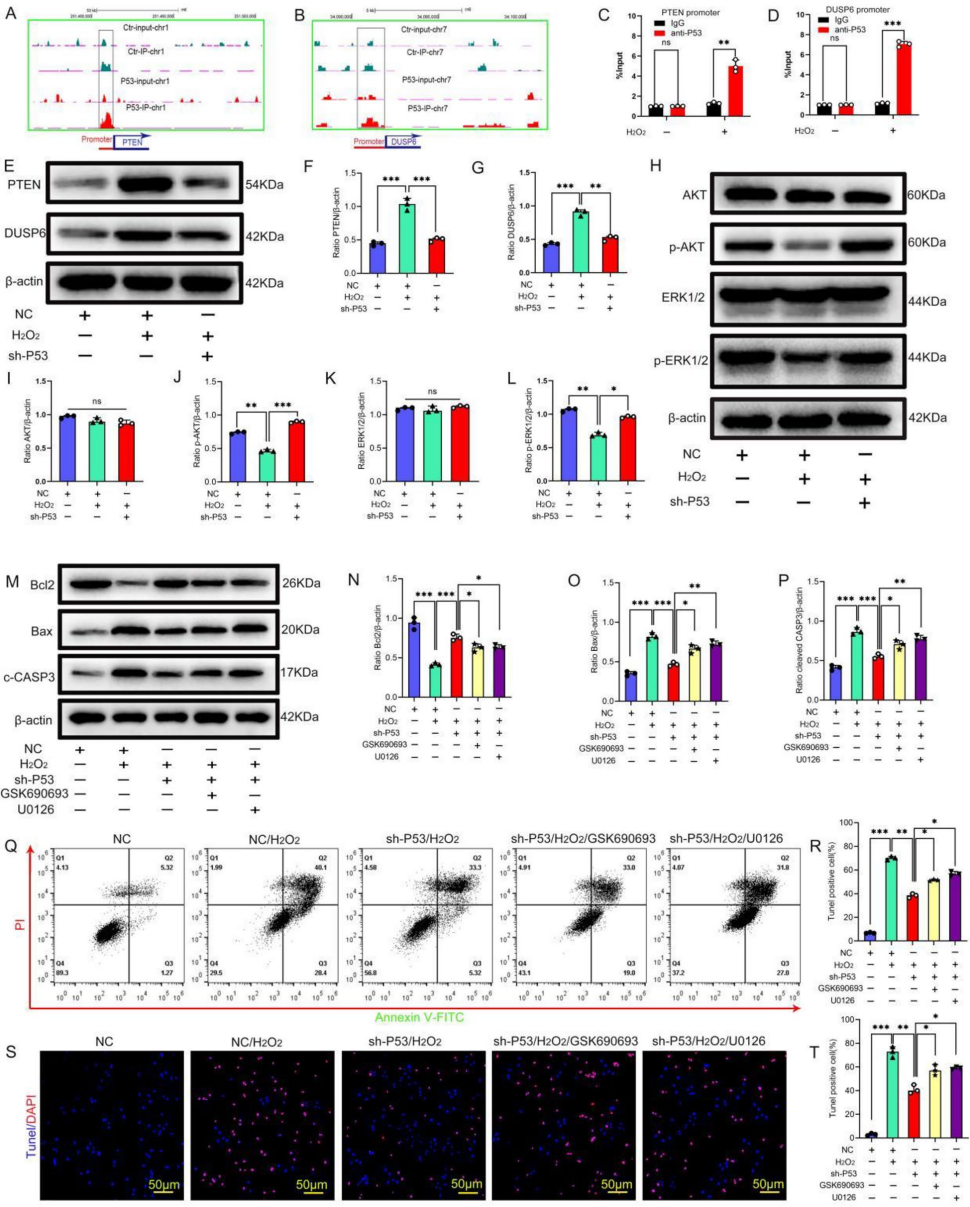
P53 mediates stress-induced apoptosis in BMSCs by inhibiting the PI3K/AKT and MEK/ERK1/2 pathways. **A, B** Visualization of P53 enrichment on chromosomes 7 and 1. **C, D** ChIP-qPCR verified P53 that binds to the *DUSP6* and *PTEN* promoters. **E–G** The protein expression levels of DUSP6 and PTEN were determined by Western blot (*n* = 3). **H–L** The expression levels of AKT, p-AKT, ERK1/2, and p-ERK1/2 were determined by Western blot (*n* = 3). **M–P** The expression levels of Bcl2, Bax, and cleaved caspase-3 (C-CASP3) were determined by Western blot (*n* = 3). **Q, R** Annexin V-FITC/PI staining was conducted to analyze apoptosis (*n* = 3). **S, T** TUNEL/DAPI staining was conducted to determine the number of TUNEL-positive cells (*n* = 3). In **C, D, F, G, I–L, N–P, R, T**, data are presented as mean ± standard deviation, and statistical analysis was performed using one-way ANOVA and the Tukey post hoc test

To further investigate how P53 accumulation under oxidative stress regulates downstream pathways mediating apoptosis in BMSCs, ChIP-seq analysis of P53 response elements was conducted. The results show that P53 was enriched in the promoter regions of *PTEN* and *DUSP6*, and oxidative stress significantly enhanced P53 enrichment in the promoter regions of *PTEN* and *DUSP6* (Fig. 5A, B). Subsequent ChIP-qPCR validation of the enrichment results showed that P53 interacted with the *PTEN* and *DUSP6* promoters (Fig. 5C, D). Next, the expression of PTEN and DUSP6 in BMSCs under oxidative stress and the effect of P53 knockdown in BMSCs on PTEN and DUSP6 expression were determined. The results show that PTEN and DUSP6 levels in the oxidative stress group were significantly increased compared with the NC group. Knockdown of P53 significantly suppressed the expression of PTEN and DUSP6 (Fig. 5E–G). In conclusion, P53 can regulate the expression of PTEN and DUSP6.

PTEN inhibits PI3K/AKT signaling by converting PIP3 to PIP2. DUSP6, a member of the MAPK family, specifically interacts with ERK1/2 and inhibits its activity [26, 27]. P53 may regulate the PI3K/AKT and MEK/ERK1/2 pathways by regulating the expression of PTEN and DUSP6. The protein expression levels of AKT, p-AKT, ERK1/2, and p-ERK1/2 were determined in P53 knockdown BMSCs. The results showed that compared with the empty vector group, the p-AKT and p-ERK1/2 levels were significantly increased in the P53 knockdown group (Fig. 5H–L), indicating that P53 knockdown promotes the activation of the PI3K/AKT and MEK/ERK1/2 pathways. Finally, we verified whether P53 mediates apoptosis in BMSCs through the PI3K/AKT and MEK/ERK1/2 pathways. We treated BMSCs with the PI3K/AKT pathway-specific inhibitor GSK690693 (30 μM, 72 h) or the MEK/ERK1/2 pathway-specific inhibitor U0126 (10 μM, 2 h). The results showed that knockdown of P53 could significantly inhibit oxidative stress-induced apoptosis in BMSCs. After the addition of specific inhibitors of the PI3K/AKT pathway and the MEK/ERK1/2 pathway, the expression of pro-apoptotic proteins in BMSCs was significantly upregulated and the expression of anti-apoptotic proteins was significantly downregulated. The apoptosis rate of BMSCs was significantly increased (Fig. 5M–T), indicating that specific inhibitors of the PI3K/AKT pathway and the MEK/ERK1/2 pathway reversed the effect of P53 knockdown on oxidative stress-induced apoptosis in BMSCs. In conclusion, P53 can mediate stress-induced apoptosis in BMSCs by inhibiting the PI3K/AKT and MEK/ERK1/2 pathways.

## Discussion

**Fig. 6:**
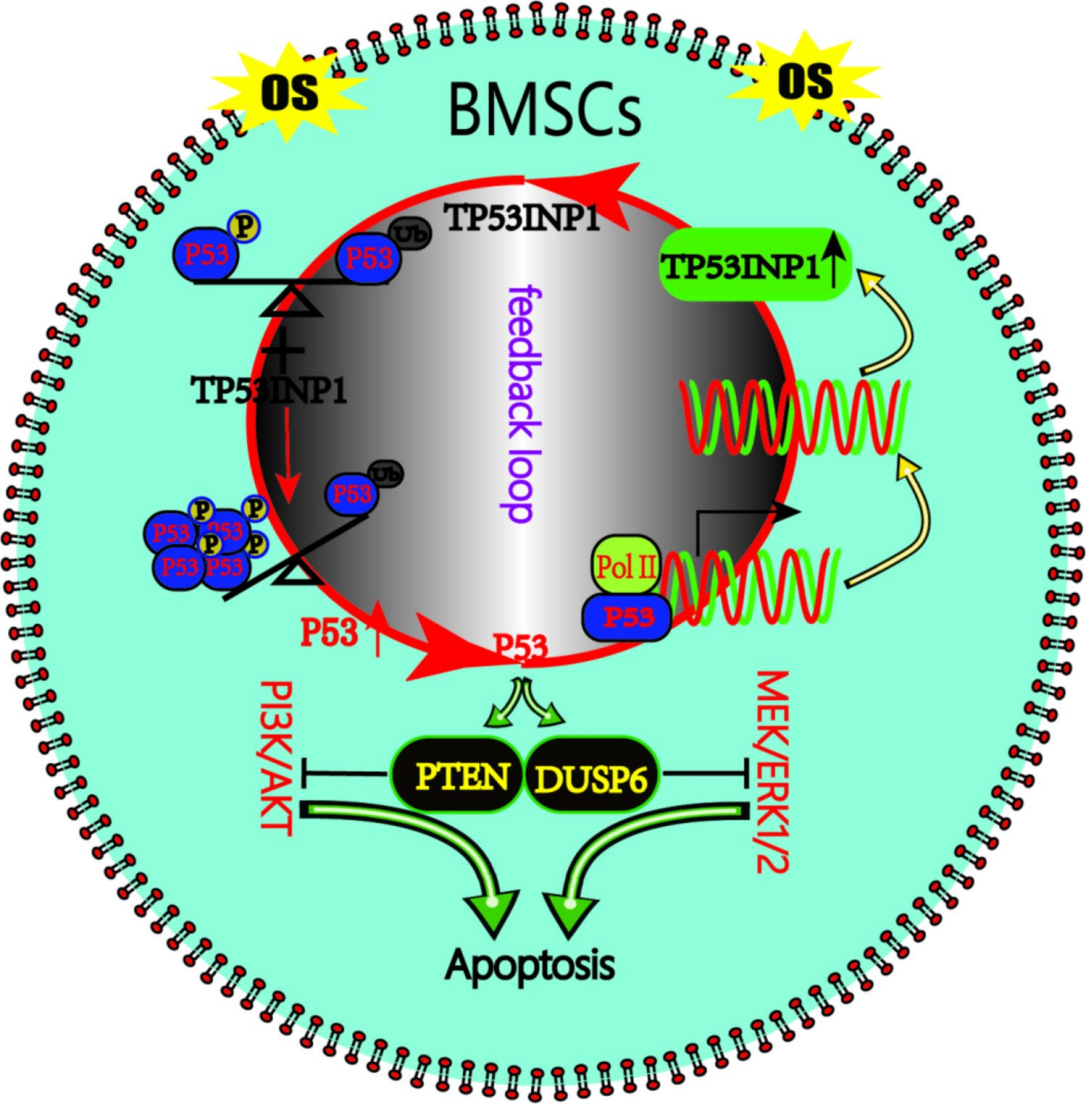
Under oxidative stress, TP53INP1 and P53 form a positive feedback loop to regulate apoptosis in BMSCs. TP53INP1 and P53 regulate each other under oxidative stress. TP53INP1 increases the phosphorylation of P53, inhibits the ubiquitination and degradation of P53, and promotes the accumulation of P53 in BMSCs. P53 in turn binds to the *TP53INP1* promoter and enhances *TP53INP1* transcription. Under oxidative stress, the two form a positive feedback loop, which further inhibits the PI3K/Akt and MEK/ERK1/2 pathways to promote stress-induced apoptosis in BMSCs

In the present study, we explored the mechanism by which the TP53INP1–P53 positive feedback loop mediates apoptosis in BMSCs in an oxidative stress-induced cell injury model. TP53INP1 promotes P53 accumulation in BMSCs by increasing P53 phosphorylation and inhibiting P53 ubiquitination and degradation. P53 in turn binds to the *TP53INP1* promoter and enhances transcription. Under oxidative stress, the two form a positive feedback loop, which further inhibits the PI3K/AKT and MEK/ERK1/2 pathways to aggravate oxidative stress-induced apoptosis in BMSCs. This study reveals the role of the TP53INP1–P53 positive feedback loop in BMSCs (Fig. 6), which is expected to provide a new target for the prevention of oxidative stress-induced apoptosis in BMSCs.

Oxidative stress-induced apoptosis is common, and BMSCs are no exception. The oxidative stress microenvironment can lead to the accumulation of a large amount of ROS in BMSCs in the transplantation area, which activates apoptosis-related pathways and leads to apoptosis of BMSCs in the transplantation area [29, 30]. However, the specific mechanism of oxidative stress-induced BMSC apoptosis is still unclear. Induction of apoptosis is one of the core activities of P53 to exert its tumor suppressor function. *TP53INP1* is a P53-inducible gene, and TP53INP1 and P53 induce apoptosis in many tumor cell lines, but little is known about their roles in normal BMSCs [31–35]. In the present study, we found significant upregulation of TP53INP1 and P53 expression in a cell model of oxidative stress-induced BMSCs. Furthermore, in BMSCs without oxidative stress, overexpression of TP53INP1 and P53 also promoted apoptosis. Our study extends the clinical application of TP53INP1 and P53 by providing a target to prevent apoptosis in transplanted BMSCs. Oxidative stress is considered one of the important causes of BMSC apoptosis. In the oxidative stress-induced BMSC apoptosis model, we confirmed that knockdown of TP53INP1 or P53 significantly inhibits apoptosis. TP53INP1 and P53 play important roles in oxidative stress-induced apoptosis in BMSCs. However, the relationship between the two needs to be further explored. Next, we knocked down P53 in BMSCs overexpressing TP53INP1, and we found that P53 knockdown blocked the apoptosis induction effect of TP53INP1 overexpression. At the same time, overexpression of P53 in BMSCs with TP53INP1 knockdown could weaken the inhibitory effect of TP53INP1 knockdown on apoptosis. These results indicate that P53 is a downstream regulatory target of TP53INP1 and that P53 plays a dominant role in the process of oxidative stress-induced apoptosis in BMSCs.

TP53INP1 and P53 play important roles in the process of oxidative stress-induced apoptosis in BMSCs. Abnormal expression of TP53INP1 changed P53 protein expression levels in BMSCs, and abnormal expression of P53 in turn changed TP53INP1 mRNA and protein expression levels in BMSCs. TP53INP1 and P53 expression was significantly increased under oxidative stress. The two form a positive feedback loop under oxidative stress. This is the first report showing that TP53INP1 and P53 form a positive feedback loop. To determine how TP53INP1 affects P53 protein expression, we used a protein synthesis inhibitor (CHX) after knocking down TP53INP1. CHX had no effect on the P53 protein level, indicating that TP53INP1 does not affect P53 protein synthesis. Then, the proteasome inhibitor MG132 was used after knocking down TP53INP1. MG132 reversed the effect of TP53INP1 knockdown on P53 degradation. P53 degradation is mainly regulated by the ubiquitin–proteasome system [36]. TP53INP1 decreases the ubiquitination of P53 by increasing P53 phosphorylation. We set out to investigate how the accumulation of P53 contributes to the increased expression of TP53INP1 at the mRNA and protein levels. We considered that P53 regulates the expression of TP53INP1 at the transcriptional level and predicted the P53 binding sites in the *TP53INP1* promoter. ChIP-qPCR and luciferase reporter assays were used to double verify the binding of P53 to the *TP53INP1* promoter to promote its transcription. In conclusion, we found a TP53INP1–P53 positive feedback loop under oxidative stress and identified the mechanism of mutual promotion of TP53INP1 and P53 expression. The cascade reaction of the positive feedback loop amplifies the induction of apoptosis in BMSCs. The TP53INP1–P53 positive feedback loop plays a crucial role in the process of oxidative stress-induced apoptosis in BMSCs.

The present study also has some shortcomings. Transcriptome data analysis revealed that hundreds of factors were differentially expressed under oxidative stress. Although the positive feedback loop formed by TP53INP1 and P53 had a significant effect on BMSC apoptosis, TP53INP1 and P53 are not the only factors that play a role. Our results show that knockdown of either P53 or TP53INP1 could not completely alleviate oxidative stress-induced apoptosis in BMSCs. These results indicate that there are many regulatory targets affecting BMSC apoptosis under oxidative stress conditions. TP53INP1 may play a pro-apoptotic role independent of P53; we observed that overexpression of TP53INP1 promoted apoptosis in BMSCs and knockdown of P53 in BMSCs overexpressing TP53INP1 could only partially alleviate TP53INP1-induced apoptosis. It has been found that when TP53INP1 is overexpressed in P53-deficient cells, it can inhibit cell growth and promote apoptosis by activating P73 [37]. In addition, TP53INP1 can induce cell death through the autophagy pathway [38, 39]. The mechanism by which TP53INP1 acts independently remains to be explored in depth. We further explored the role of the TP53INP1–P53 positive feedback loop in the regulation of stress-induced apoptosis in BMSCs. Our next step will be to verify the effect of this positive feedback loop on stress-induced apoptosis in BMSCs in rats.

The specific mechanism of oxidative stress-induced apoptosis of BMSCs is still unclear. We found that the key regulatory proteins TP53INP1 and P53 were upregulated in the oxidative stress-induced apoptosis model of BMSCs by transcriptome sequencing, which further confirmed that TP53INP1 and P53 form a positive feedback loop to regulate BMSC apoptosis under oxidative stress conditions. The positive feedback loop of TP53INP1–P53 was first confirmed in an oxidative stress-induced apoptosis model of BMSCs. This positive feedback loop is expected to serve as a new target to prevent oxidative stress-induced apoptosis in BMSCs.

## Methods

### Experimental animals

BMSCs were isolated from 10 young SPF-grade Sprague–Dawley rats (male or female) weighing 20–30 g. All animal experiments were approved by the Experimental Animal Ethics Committee of Guizhou Medical University (Ethical review No. 2200603).

### Isolation and culture of BMSCs

Rats were sacrificed and bilateral femurs and tibias were harvested under sterile conditions. The medullary cavity was rinsed with complete L-DMEM medium supplemented with 10% fetal bovine serum (FBS; Gibco, Carlsbad, CA, USA) and 1% double antibody (Invitrogen, Carlsbad, CA, USA) to obtain bone marrow tissue. After centrifugation at 1000 rpm for 5 min, bone marrow tissue was collected. The bone marrow tissue was resuspended in complete L-DMEM medium, fully resuspended, inoculated into cell culture flasks, and cultured at 37 °C in 5% CO_2_. When the primary BMSCs reached 80%–90% confluence, the cells were treated with 0.25% trypsin–0.02% ethylenediamine tetraacetic acid (EDTA) (Gibco, Carlsbad, CA, USA) and split 1:3. BMSCs of the third passage were used for experiments.

### Osteogenic differentiation of BMSCs

BMSCs of the third passage were seeded into six-well plates. When the cell confluence reached 60%–70%, the medium in the experimental group was replaced by BMSC osteogenic induction medium according to the BMSC osteogenic induction kit (Cyagen Biosciences, USA), and the medium in the control group was unchanged. After 2 weeks of osteogenic induction, cells were fixed and ALP activity was determined by staining with alkaline phosphatase (ALP) (Cyagen Biosciences). Calcium nodules were identified by staining with 0.1% alizarin red (Cyagen Biosciences).

### Adipogenic differentiation of BMSCs

BMSCs of the third passage were seeded into six-well plates. When the cell confluence reached 90%, the medium in the experimental group was replaced by BMSC adipogenic induction medium according to the BMSC adipogenic induction kit (Cyagen Biosciences), and the medium in the control group was unchanged. After 3 weeks of adipogenic induction, lipid droplets were identified by oil red O (Cyagen Biosciences) staining.

### Chondrogenic differentiation of BMSCs

BMSCs of the third passage were seeded into six-well plates. When the cell confluence reached 60%, the medium in the experimental group was replaced with BMSC chondrogenic induction medium according to the BMSC chondrogenic induction kit (Cyagen Biosciences), and the medium in the control group was unchanged. After 4 weeks of chondrogenic induction, cartilage acidic mucopolysaccharides were detected by alcian blue (Cyagen Biosciences) staining.

### Identification of BMSCs

The density of passage 3 BMSCs was adjusted to 2 × 10^7^ cells/mL, and 50 µL of cell suspension was used per sample. Staining buffer was used in the control group. Cells were incubated with 5 µL mouse anti-CD34-FITC (BD, USA), mouse anti-CD45-FITC (BD, USA), mouse anti-CD45-FITC (BD, USA), mouse anti-CD90-PECyTM7 (BD, USA), or mouse anti-CD105-PE (BD, USA). Then, 45 µL staining buffer was added to each tube, followed by mixing, and 5 µL of each antibody was put into the flow detection tube. Then, 30 µL staining buffer was added. Next, 50 µL of cell suspension was added to each tube, samples were mixed, incubated at 4 °C in the dark for 30 min, and washed three times with staining buffer, and then 500 µL staining buffer was added to each tube. Surface antigens were detected by flow cytometry (Beckman, USA).

### ***BMSCs were treated with H***_***2***_***O***_***2***_*** to establish an oxidative stress cell model***

In a previous study by our group, H_2_O_2_ was used to simulate the oxidative stress microenvironment, and an oxidative stress cell model was successfully constructed [28]. In brief, when cell confluence reached 80%, BMSCs were treated with L-DMEM complete medium with 1000 μM H_2_O_2_ for 24 h at 37 °C and 5% CO_2_ to establish the oxidative stress cell injury model.

### ROS detection

The third generation of BMSCs was seeded into laser confocal culture dishes. When the cell confluence reached 80–90%, the cells were treated with H_2_O_2_ for 24 h, washed with PBS buffer, and stained using a DCFH-DA fluorescent probe kit according to the manufacturer’s instructions (Sigma, Germany). Subsequently, 400 μL of detection solution was added, and cells were incubated for 30 min. After washing the cells with PBS, an anti-fluorescence quencher was added, and the fluorescence intensity was analyzed using laser confocal microscopy.

### H2O2 detection

The third generation of BMSCs was seeded into six-well plates. When the confluence degree of the cells reached 80–90%, they were treated with H_2_O_2_ for 24 h, washed with PBS buffer, lysed with RIPA, and processed according to the instructions of an H_2_O_2_ detection kit (Biyuntian, Shanghai, China). Finally, the absorbance OD value of each group was measured using a microplate reader at 560 nm, and the concentration of H_2_O_2_ was calculated according to the standard curve.

### Detection of superoxide anion

The third generation of BMSCs was seeded into six-well plates. When the confluence degree of the cells reached 80–90%, they were treated with H_2_O_2_ for 24 h, washed with PBS buffer, and lysed with RIPA to prepare a cell suspension. The NBT solution was prepared by adding 1 mg of NBT powder (Solebro, Beijing, China) to 10 mL of PBS and then filtering it with a 0.2-mm filter. The cell suspension and NBT solution were incubated for 40 min, followed by the addition of 120 μL of NaOH and 140 μL of DMSO. Finally, the absorbance OD value of each group was determined using a microplate reader at 630 nm.

### MDA detection

The third generation of BMSCs was seeded into six-well plates. When the confluence degree of the cells reached 80–90%, they were treated with H_2_O_2_ for 24 h, washed with PBS buffer, lysed with RIPA, and processed according to the instructions of an MDA detection kit (Biyuntian, Shanghai, China). Finally, the absorbance OD value of each group was measured using a microplate reader at 532 nm.

### SOD detection

The third generation of BMSCs was seeded into six-well plates. When the confluence degree of the cells reached 80–90%, they were treated with H_2_O_2_ for 24 h, washed with PBS buffer, lysed with RIPA, and processed according to the instructions of the SOD activity detection kit Biyuntian, Shanghai, China). Finally, the absorbance OD value of each group was measured using a microplate reader at 560 nm.

### RNA sequencing (RNA-seq)

Total RNA was extracted by TRIzol. RNA was quantified and RNA purity was determined by a NanoDrop 2000 spectrophotometer. RNA integrity was determined by an Agilent 2100 bioanalyzer. mRNA with poly-A tails was enriched by Oligo(dT) magnetic beads and rRNA was removed from the total RNA to obtain mRNA. mRNAs were randomly interrupted with divalent cations in NEB Fragmentation Buffer, and libraries were constructed using the NEBNext® Ultra™ RNA Library Prep Kit for Illumina® kit (NEB, China). Transcriptome sequencing and analysis were performed by Shanghai Jikai Gene Medical Technology Co., Ltd. After quality control of sequencing data, the clean reads were aligned with a rat reference genome using HISAT2 software, and the htseq-count tool was used to calculate the gene expression level according to the alignment results. The differential expression results were obtained by DESeq2. Genes with |log_2_(fold change)|≥ 1 and *P* < 0.05 were considered as differentially expressed genes.

### Lentivirus transfection

All overexpression and knockdown lentiviruses were purchased from Shanghai Jikai Gene Medical Technology Company, Ltd. The optimal MOI (MOI = 80) and optimal transfection conditions (En.S + Polybrane) were found through preliminary experiments before transfection. BMSCs were infected with lentivirus to establish a blank control group. After 12 h, the medium was replaced by complete L-DMEM. After 72 h of infection, the stable cells were screened by adding 2 µg/mL puromycin. Finally, RNA and protein were extracted and gene expression was detected.

### Real-time qPCR

Total RNA was extracted using TRIzol, and the concentration and purity of RNA were determined by a Nanodrop 2000 spectrophotometer. The reaction system was configured with Oligo (dT) primers and M-MuLV reverse transcriptase, and the reaction time and temperature were set according to the instructions of the kit. The cDNA was then used as a template for PCR amplification on a real-time fluorescence quantitative PCR instrument. Relative mRNA expression was calculated using the 2^−ΔΔCt^ method. The primer sequences used in this study were as follows:

$$ \begin{gathered} {\text{P53}} - {\text{F}}:{\text{ CCCCTGAAGACTGGATAACTGT}}; \, \hfill \\ {\text{P53}} - {\text{R}}:{\text{ ATTAGGTGACCCTGTCGCTG}}; \, \hfill \\ {\text{TP53INP1}} - {\text{F}}:{\text{ CAACAACAAAAGGACCCGGAC}}; \, \hfill \\ {\text{TP53INP1}} - {\text{R}}:{\text{ GAGTCATCATCCGTGAGCCG}}; \, \hfill \\ {\text{PTEN}} - {\text{F}}:{\text{GGAAAGGACGGACTGGTGTA}}; \hfill \\ {\text{PTEN}} - {\text{R}}:{\text{ TACATAGCGGCCTCTGACTGG}}; \, \hfill \\ {\text{DUSP6}} - {\text{F}}:{\text{ CAGTGGTGCTCTACGACGAG}}; \, \hfill \\ {\text{DUSP6}} - {\text{R}}:{\text{ GCAATGCAGGGAGAACTCGGC}}; \, \hfill \\ {\text{GAPDH}} - {\text{F}}:{\text{ GACATGCCGCCTGGAGAAAC}}; \hfill \\ {\text{GAPDH}} - {\text{R}}:{\text{ AGCCCAGGATGCCCTTTAGT}} \hfill \\ \end{gathered} $$

### Western blot

Cells were lysed with RIPA cell lysis buffer (Solebro, Beijing, China) and proteins were extracted. A BCA protein quantification kit (Biyuntian, Shanghai, China) was used for protein quantification. Proteins were separated by SDS-PAGE and transferred to PVDF membranes (Millipore, Billerica, MA, USA). The membranes were then blocked with blocking solution (5% skimmed milk powder solution) at room temperature for 1 h and incubated overnight at 4 °C with rabbit anti-β-actin (4970, Cell Signaling), mouse anti-P53 (1:1000, ab26, Abcam), rabbit anti-TP53INP1 (1:2000, ab202026, Abcam), rabbit anti-Cleaved Caspase-3 (1:1000, ab32042, Abcam), rabbit anti-Bcl-2 (1:1500, ab196495, Abcam), rabbit anti-Bax (1:2000, 14,796, Cell Signaling), rabbit anti-AKT (1:1000, ab8805, Abcam), rabbit anti-AKT (phospho T308) (1:1500, ab38449, Abcam), rabbit anti-ERK1 + ERK2 (1:5000, ab184699, Abcam), rabbit anti-ERK1 (phospho T202) + ERK2 (phospho T185) (1:1000, ab201015, Abcam), rabbit anti-PTEN (1:1000, ab267787, Abcam), rabbit anti-DUSP6 (1:500, ab76310, Abcam). The membranes were washed and incubated with HRP-linked anti-mouse IgG (1:3000, 7076, Cell Signaling) and HRP-linked anti-rabbit IgG (1:3000, 7074, Cell Signaling) for 1 h at room temperature. Protein bands were visualized with the ECL luminescence kit (Pierce, Rockford, IL, USA), and images were acquired using a gel imaging system and analyzed using ImageJ (National Institutes of Health, Bethesda, MD, USA).

### Flow cytometry

The cell culture medium was collected, the adherent cells were digested with trypsin without EDTA, the collected culture medium was mixed with the cell digest, the cell precipitate was collected by centrifugation, the precipitate was resuspended in 1 × Binding Buffer, and 100 µL of the cell suspension was transferred to a 5-mL flow tube. Using the Annexin V-FITC Apoptosis detection kit (BD Biosciences, San Jose, California, USA), we directly added 5 μL Annexin V-FITC and 5 μL PI according to the manufacturer’s instructions. Cells were gently vortexed and incubated in the dark for 20 min at room temperature. We then analyzed apoptosis by flow cytometry (Beckman Coulter, Indianapolis, Indiana, USA).

### TUNEL staining

Adherent cells were washed three times with PBS, fixed with 4% neutral paraformaldehyde for 30 min at room temperature, and permeabilized with 0.3% Triton X-100. TUNEL assay solution (Biyuntian, Shanghai, China) was added, and samples were incubated in the dark for 1 h. Subsequently, the cells were stained with DAPI (Solebro, Beijing, China) for 4 min and washed with PBS, the samples were sealed with an anti-fluorescence quench agent, and fluorescence was observed by laser confocal microscopy (Zeiss, Germany).

### Luciferase reporter assay

Plasmids were purchased from Shanghai Jikai Gene Medical Technology Company, Ltd. The adherent cells were digested, collected, and centrifuged, the culture medium was decanted, and the cells were counted after washing. The cell density was adjusted to 1 × 10^6^ cells/mL, and the cells were seeded in 12-well plates at a density of 1 × 10^5^ cells/well and cultured for 24 h. According to the instructions of the dual luciferase reporter gene detection kit (Biyuntian, Shanghai, China), the dual luciferase plasmid was added, serum-free DMEM and Lipofectamin2000 were mixed in another 1.5-mL EP tube, and the plasmid and reagent were mixed. After the culture solution was discarded, the transfection mixture was gradually added to the 24-well plate. After mixing, the samples were incubated for 5 h. Then, 500 μL DMEM medium containing 10% FBS was added and cells were cultured for another 24 h. Finally, luciferase activity was detected by a multifunctional enzyme labeling system (BioTek) at 465 nm.

### ChIP

Cellular chromatin was cross-linked using 37% formaldehyde. The cells were incubated for 15 min at room temperature. The cross-linking reaction was terminated by glycine. Cells were lysed by adding SDS lysis buffer after washing with PBS, and chromatin was disrupted by sonication. After centrifugation, the supernatant was collected. Next, 200μL of supernatant was diluted, added to protein A-salmon sperm DNA AGAR, and incubated at 4 °C for 1 h. After centrifugation, the supernatant was removed and 20μL was used as input DNA. The supernatant was added to the corresponding antibody and negative control Ig. The mixture was turned and mixed overnight at 4 °C. Protein A-salmon sperm DNA AGAR was added, the mixture was turned over and incubated for 1 h at 4 °C, chromatin was eluted, cross-linking was deactivated, and DNA in the immunoprecipitate was extracted. DNA sequencing and analysis were performed by Shanghai Jikai Gene Medical Technology Company, Limited. The enrichment of transcription factors in the promoter region was analyzed by PCR amplification using the corresponding promoter-specific primers.

### Co-immunoprecipitation

BMSCs of the third passage were taken, and after treatment, when the cells reached 70%–90% confluence, the proteins were extracted. The cells were washed three times with PBS buffer with shaking for 3 min each time, NP40 (Solarbio, Beijing, China) was added to lyse the cells, and the cell lysate was sonicated with an ultrasonic crusher. After centrifugation, the supernatant was removed, 100μL of cell lysate was used as input sample, and the remaining cell lysate was used for immunoprecipitation. Cell lysates were pretreated with Protein A/G agarose beads (Solarbio, Beijing, China), nonspecific binding proteins were removed from lysates, and the supernatant was collected by centrifugation. P53-specific antibodies were added to the supernatant and incubated overnight at 4 °C. Protein A/G agarose beads were pretreated with lysis buffer, Protein A/G agarose beads were added, and samples were incubated for 2 h at 4 °C with rotation. After centrifugation, the supernatant was removed, the immune complexes were washed, loading buffer was added, antigens, antibodies, and agarose beads were dissociated by boiling, and the supernatant was collected by centrifugation. P53 ubiquitination was analyzed by Western blot.

### Statistical analysis

All data were analyzed using SPSS 26.0, and the data were plotted using GraphPad Prism 8 (GraphPad Software, San Diego, California, USA). Parametric or non-parametric statistical tests were selected by determining first if the data were normally distributed using the Shapiro–Wilk normality test. If the data follow a normal distribution and their variance is uniform, they are presented as mean ± standard deviation (SD). Statistical significance was determined using the two-tailed unpaired Student’s *t*-test for comparison between two groups or by one-way ANOVA followed by Tukey’s post hoc test for multiple comparisons (more than two groups). If the data do not follow a normal distribution, they are presented as M(P25, P75), and the Kruskal–Wallis rank-sum test was used for comparisons between groups. If the difference between groups was statistically significant, the Dwass–Steel–Critchlow–Fligner method was further used for multiple comparisons. *P* < 0.05 was considered statistically significant.

### Supplementary Information

Below is the link to the electronic supplementary material.

Supplementary file1 (DOCX 494 kb)

## Data Availability

The datasets during and/or analyzed during the current study available from the corresponding author on reasonable request.

## References

[1] Edgar L, Pu T, Porter B, Aziz JM, La Pointe C, Asthana A, Orlando G (2020). Regenerative medicine, organ bioengineering and transplantation. Br J Surg.

[2] Guo Y, Jia X, Cui Y, Song Y, Wang S, Geng Y, Li R, Gao W, Fu D (2021). Sirt3-mediated mitophagy regulates AGEs-induced BMSCs senescence and senile osteoporosis. Redox Biol.

[3] Ahuja CS, Nori S, Tetreault L, Wilson J, Kwon B, Harrop J, Choi D, Fehlings MG (2017). Traumatic spinal cord injury-repair and regeneration. Neurosurgery.

[4] Guo Y, Yu Y, Hu S, Chen Y, Shen Z (2020). The therapeutic potential of mesenchymal stem cells for cardiovascular diseases. Cell Death Dis.

[5] Lee S, Choi E, Cha MJ, Hwang KC (2015). Cell adhesion and long-term survival of transplanted mesenchymal stem cells: a prerequisite for cell therapy. Oxid Med Cell Longev.

[6] Denu RA, Hematti P (2016). Effects of oxidative stress on mesenchymal stem cell biology. Oxid Med Cell Longev.

[7] Zhang F, Yan Y, Peng W, Wang L, Wang T, Xie Z, Luo H, Zhang J, Dong W (2021). PARK7 promotes repair in early steroid-induced osteonecrosis of the femoral head by enhancing resistance to stress-induced apoptosis in bone marrow mesenchymal stem cells via regulation of the Nrf2 signaling pathway. Cell Death Dis.

[8] Wang T, Zhang F, Peng W, Wang L, Zhang J, Dong W, Tian X, Ye C, Li Y, Gong Y (2022). Overexpression of NMNAT3 improves mitochondrial function and enhances antioxidative stress capacity of bone marrow mesenchymal stem cells via the NAD+-Sirt3 pathway. Bioscience reports.

[9] Zhang B, Pan C, Feng C, Yan C, Yu Y, Chen Z, Guo C, Wang X (2022). Role of mitochondrial reactive oxygen species in homeostasis regulation. Redox Report Commun Radical Res.

[10] Sinha K, Das J, Pal PB, Sil PC (2013). Oxidative stress: the mitochondria-dependent and mitochondria-independent pathways of apoptosis. Arch Toxicol.

[11] Sies H (2015). Oxidative stress: a concept in redox biology and medicine. Redox Biol.

[12] Beyfuss K, Hood DA (2018). A systematic review of p53 regulation of oxidative stress in skeletal muscle. Redox Rep Commun Free Radical Res.

[13] Liu Y, Xu W, Zhai T, You J, Chen Y (2019). Silibinin ameliorates hepatic lipid accumulation and oxidative stress in mice with non-alcoholic steatohepatitis by regulating CFLAR-JNK pathway. Acta Pharmaceutica Sinica BB.

[14] Xiao Q, Werner J, Venkatachalam N, Boonekamp KE, Ebert MP, Zhan T (2022). Cross-talk between p53 and Wnt signaling in cancer. Biomolecules.

[15] Matsuda S, Nakagawa Y, Kitagishi Y, Nakanishi A, Murai T (2018). Reactive oxygen species, superoxide dimutases, and PTEN-p53-AKT-MDM2 signaling loop network in mesenchymal stem/stromal cells regulation. Cells.

[16] Yang Y, Zhang M, Zhang Y, Liu K, Lu C (2023). 5-Fluorouracil suppresses colon tumor through activating the p53-fas pathway to sensitize myeloid-derived suppressor cells to FasL+ cytotoxic t lymphocyte cytotoxicity. Cancers.

[17] Revathidevi S, Munirajan AK (2019). Akt in cancer: mediator and more. Semin Cancer Biol.

[18] Shi T, van Soest DMK, Polderman PE, Burgering BMT, Dansen TB (2021). DNA damage and oxidant stress activate p53 through differential upstream signaling pathways. Free Radical Biol Med.

[19] Yang J, Maity B, Huang J, Gao Z, Stewart A, Weiss RM, Anderson ME, Fisher RA (2013). G-protein inactivator RGS6 mediates myocardial cell apoptosis and cardiomyopathy caused by doxorubicin. Can Res.

[20] Nagpal I, Yuan ZM (2021). The basally expressed p53-mediated homeostatic function. Front Cell Develop Biol.

[21] Saadi H, Seillier M, Carrier A (2015). The stress protein TP53INP1 plays a tumor suppressive role by regulating metabolic homeostasis. Biochimie.

[22] Cai Q, Zeng S, Dai X, Wu J, Ma W (2017). miR-504 promotes tumour growth and metastasis in human osteosarcoma by targeting TP53INP1. Oncol Rep.

[23] Cano CE, Gommeaux J, Pietri S, Culcasi M, Garcia S, Seux M, Barelier S, Vasseur S, Spoto RP, Pébusque MJ, Dusetti NJ, Iovanna JL, Carrier A (2009). Tumor protein 53-induced nuclear protein 1 is a major mediator of p53 antioxidant function. Can Res.

[24] Shahbazi J, Lock R, Liu T (2013). Tumor protein 53-induced nuclear protein 1 enhances p53 function and represses tumorigenesis. Front Genet.

[25] Aubrey BJ, Kelly GL, Janic A, Herold MJ, Strasser A (2018). How does p53 induce apoptosis and how does this relate to p53-mediated tumour suppression?. Cell Death Differ.

[26] Chen YH, Yang SF, Yang CK, Tsai HD, Chen TH, Chou MC, Hsiao YH (2021). Metformin induces apoptosis and inhibits migration by activating the AMPK/p53 axis and suppressing PI3K/AKT signaling in human cervical cancer cells. Mol Med Rep.

[27] Yue J, López JM (2020). Understanding MAPK signaling pathways in apoptosis. Int J Mol Sci.

[28] Zhang F, Peng W, Zhang J, Dong W, Yuan D, Zheng Y, Wang Z (2019). New strategy of bone marrow mesenchymal stem cells against oxidative stress injury via Nrf2 pathway: oxidative stress preconditioning. J Cell Biochem.

[29] Liu B, Gan X, Zhao Y, Gao J, Yu H (2021). Inhibition of HMGB1 reduced high glucose-induced BMSCs apoptosis via activation of AMPK and regulation of mitochondrial functions. J Physiol Biochem.

[30] Zhang F, Peng W, Zhang J, Dong W, Wu J, Wang T, Xie Z (2020). P53 and parkin co-regulate mitophagy in bone marrow mesenchymal stem cells to promote the repair of early steroid-induced osteonecrosis of the femoral head. Cell Death Dis.

[31] Roberts O, Paraoan L (2020). PERP-ing into diverse mechanisms of cancer pathogenesis: Regulation and role of the p53/p63 effector PERP. Biochim Biophys Acta Rev Cancer.

[32] Liao CY, Yang SF, Wu TJ, Chang H, Huang CF, Liu YF, Wang CH, Liou JC, Hsu SL, Lee H, Sheu GT, Chang JT (2021). Novel function of PERP-428 variants impacts lung cancer risk through the differential regulation of PTEN/MDM2/p53-mediated antioxidant activity. Free Radical Biol Med.

[33] Han L, Huang Z, Liu Y, Ye L, Li D, Yao Z, Wang C, Zhang Y, Yang H, Tan Z, Tang J, Yang Z (2021). MicroRNA-106a regulates autophagy-related cell death and EMT by targeting TP53INP1 in lung cancer with bone metastasis. Cell Death Dis.

[34] Li W, Bi C, Han Y, Tian T, Wang X, Bao H, Xu X, Zhang X, Liu L, Zhang W, Gao H, Wang H, Zhang H, Meng B, Wang X, Fu K (2019). Targeting EZH1/2 induces cell cycle arrest and inhibits cell proliferation through reactivation of p57CDKN1C and TP53INP1 in mantle cell lymphoma. Canc Biol Med.

[35] Peuget S, Bonacci T, Soubeyran P, Iovanna J, Dusetti NJ (2014). Oxidative stress-induced p53 activity is enhanced by a redox-sensitive TP53INP1 SUMOylation. Cell Death Differ.

[36] Zhu H, Gao H, Ji Y, Zhou Q, Du Z, Tian L, Jiang Y, Yao K, Zhou Z (2022). Targeting p53-MDM2 interaction by small-molecule inhibitors: learning from MDM2 inhibitors in clinical trials. J Hematol Oncol.

[37] Tomasini R, Seux M, Nowak J, Bontemps C, Carrier A, Dagorn JC, Pébusque MJ, Iovanna JL, Dusetti NJ (2005). TP53INP1 is a novel p73 target gene that induces cell cycle arrest and cell death by modulating p73 transcriptional activity. Oncogene.

[38] Liu X, Zhou Z, Wang Y, Zhu K, Deng W, Li Y, Zhou X, Chen L, Li Y, Xie A, Zeng T, Wang G, Fu B (2020). Downregulation of HMGA1 mediates autophagy and inhibits migration and invasion in bladder cancer via miRNA-221/TP53INP1/p-ERK Axis. Front Oncol.

[39] Dinh E, Rival T, Carrier A, Asfogo N, Corti O, Melon C, Salin P, Lortet S, Kerkerian-Le Goff L (2021). TP53INP1 exerts neuroprotection under ageing and Parkinson’s disease-related stress condition. Cell Death Dis.

