# Supplementary material for: A self-amplifying loop of TP53INP1 and P53 drives oxidative stress-induced apoptosis of bone marrow mesenchymal stem cells
Source: Apoptosis. 2024 Mar 16;29(5-6):882–97. doi: 10.1007/s10495-023-01934-1 (PMC11055765; doi:10.1007/s10495-023-01934-1)
Supplement: Supplementary file 1 — Supplementary file1 (DOCX 494 kb) [file 10495_2023_1934_MOESM1_ESM.docx]

Supplementary information

Cultivation and identification of BMSCs: The original method to generate BMSCs from whole bone marrow and culture the obtained cells was adopted to improve the inoculation training, training after 48 h, culture bottle bottom to see a small amount of lipid droplets and more impurities cells, a small amount of adherent cells, smaller volume, morphological heterogeneity (A1). Confluence after 7 days was 90%, and cell morphology tended to be uniform, with a long spindle and a fish-shaped arrangement (A2). BMSCs of the third passage are shown (A3). No significant changes in cell morphology were observed after resuscitation (A4). The cells were passaged 1:3, and no significant changes in morphology were observed after successive splitting.

Identification of BMSC surface markers: BMSCs of the third passage with good morphology were used to identify BMSCs by flow cytometry based on the presence/absence of cell surface antigens, including high expression of CD105 and CD90, with positive rates of 99.7% and 99.9%, respectively, and low expression of CD45 and CD34, with positive rates of 99.9% and 99.8%, respectively (1B), indicating that the cultured BMSCs met the identification criteria and the cell purity met the experimental requirements.

BMSC differentiation assay: Osteogenesis induction medium was used to induce differentiation of BMSCs of the third passage. Cells were differentiated well after 2 weeks of differentiation, as indicated by ALP staining. The results showed that a large amount of blue-purple material could be seen after staining in the experimental group, and the staining of control BMSCs cultured in conventional medium was negative (C1). BMSCs were cultured with chondrogenic induction medium for 21 days and stained with Alcian blue. The staining results showed that blue-stained proteoglycan was deposited in the experimental group, but not in the control group (C2). BMSCs were cultured with A and B adipogenic induction medium, and oil red O staining was performed. The results showed that a large number of red lipid droplets could be seen in the experimental group after oil red O staining, while staining in the control group was negative (C3). These results suggest that BMSCs have the ability of osteogenic, adipogenic, and chondrogenic differentiation.

To establish the oxidative stress model, BMSCs were treated with different concentrations of H_2_O_2_ for 24 h, and ROS(D), H_2_O_2_(E), superoxide anion (F), MDA (G), and the antioxidant enzyme SOD (H) were detected. Finally, 1000uM of H_2_O_2_ was selected for the establishment of the oxidative stress model

The effects of lentivirus transfection on the overexpression of P53, knockdown of P53, overexpression of TP53INP1, and knockdown of TP53INP1 in BMSCs were evaluated (Supplementary Figure 2).


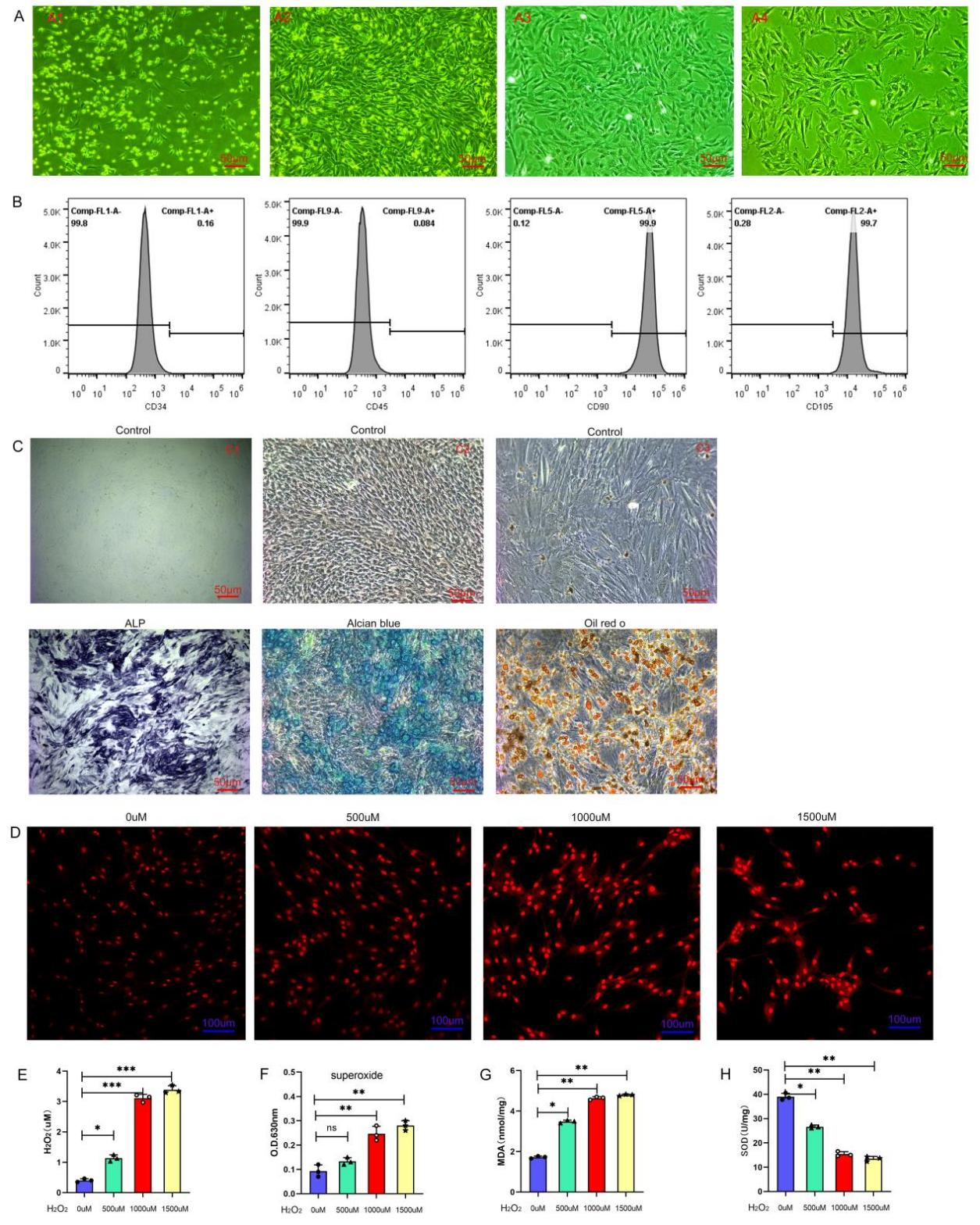


Supplementary Figure 1. Cultivation and identification of BMSCs. (A) Morphology of BMSCs at different stages. (B) Flow cytometry of BMSCs after staining for CD34, CD45, CD90, and CD105. (C) Induction of osteogenic, adipogenic, and chondrogenic differentiation of BMSCs, followed by ALP staining, Alcian blue staining, and oil red O staining,(D) DCFH-DA fluorescence detection of ROS,,(E) Intracellular H_2_O_2_ content, (F) Intracellular superoxide ion levels, (G) Intracellular MDA content, (H) Intracellular SOD viability, In (E–H), data are presented as mean ± standard deviation, and statistical analysis was conducted using one-way ANOVA and the Tukey post hoc test.


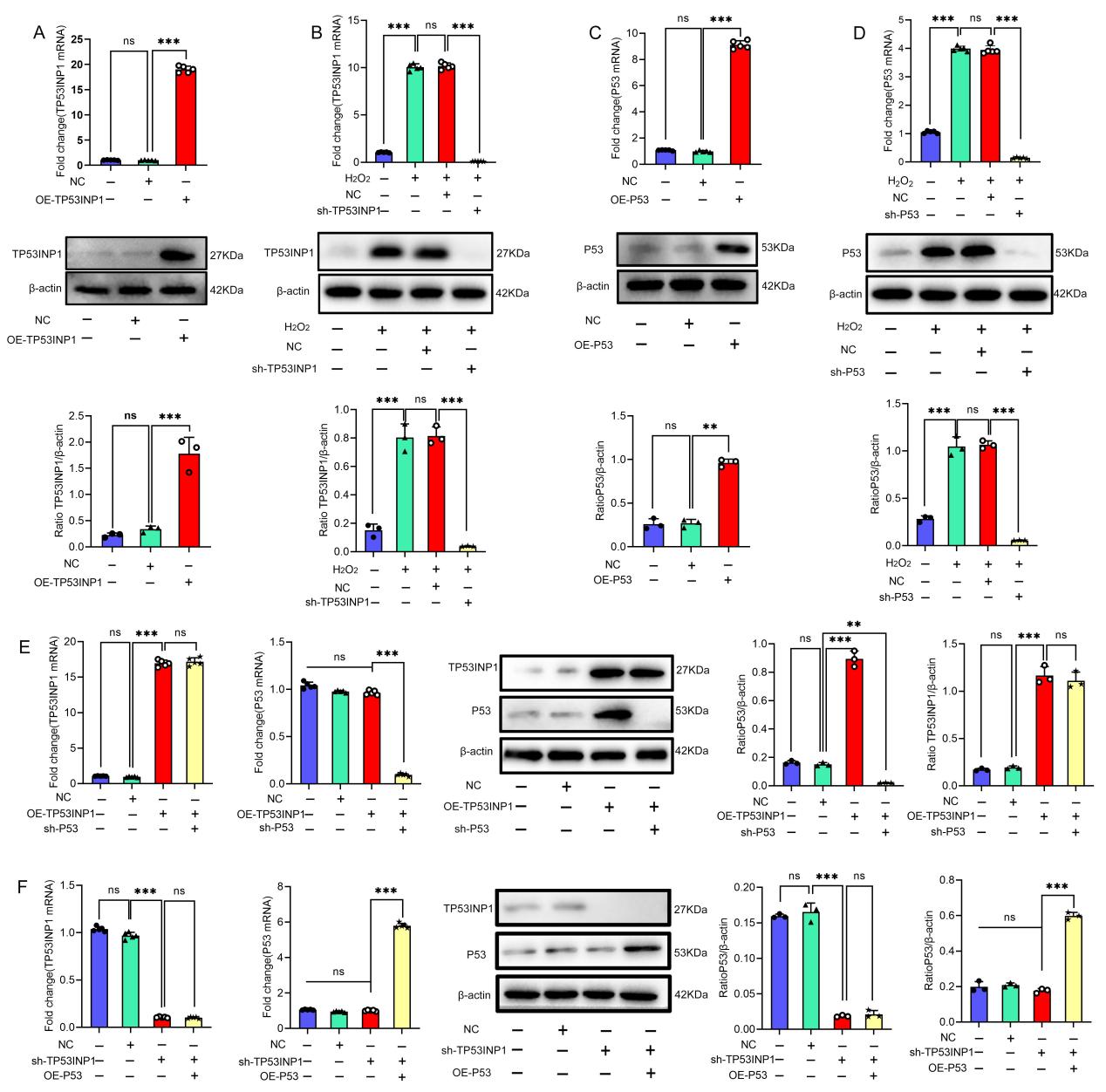


Supplementary Figure 2. Effect of lentivirus transfection of BMSCs. (A) Western blot and qPCR analyses of TP53INP1 expression. Vec, slow virus empty carrier; OE-TP53INP1, TP53INP1 overexpression. (B) Western blot and qPCR analyses of TP53INP1 knockdown efficiency. sh-TP53INP1, TP53INP1 knockdown. (C) P53 overexpression as confirmed by Western blot and qPCR. OE-P53, P53 overexpression. (D) Western blot and qPCR analyses were conducted to determine the efficiency of P53 knockdown. sh-P53, P53 knockdown. (E) Western blot and qPCR analyses were conducted to determine the efficiency of P53 knockdown in cells overexpressing TP53INP1. (F) Western blot and qPCR analyses were conducted to determine P53 overexpression efficiency in cells with TP53INP1 knockdown. In (D–I), data are presented as mean ± standard deviation, and statistical analysis was conducted using one-way ANOVA and the Tukey post hoc test.
